# Current Incidence and Risk Factors of Fecal Incontinence After Acute Stroke Affecting Functionally Independent People

**DOI:** 10.3389/fneur.2021.755432

**Published:** 2021-11-01

**Authors:** Giuseppe Lucente, Javier Corral, Luis Rodríguez-Esparragoza, Sara Castañer, Hector Ortiz, Anna Piqueras, Joaquim Broto, María Hernández-Pérez, Sira Domenech, Alicia Martinez-Piñeiro, Jordi Serra, Miriam Almendrote, David Parés, Mònica Millán

**Affiliations:** ^1^Neurology Service, Neuroscience Department, Hospital Universitari Germans Trias i Pujol, Universitat Autònoma de Barcelona, Badalona, Spain; ^2^General Surgery Department, Hospital Universitari Germans Trias i Pujol, Badalona, Spain; ^3^Institut de Diagnostic per Imatge (IDI), Hospital Universitari Germans Trias i Pujol, Badalona, Spain; ^4^Department of Project and Construction Engineering (EPC), Universitat Politècnica de Catalunya, Barcelona, Spain; ^5^Department of Engineering Design, Universitat Politècnica de Barcelona, Barcelona, Spain; ^6^Gastroenterology Department, Hospital Universitari Germans Trias i Pujol, Barcelona, Spain

**Keywords:** fecal incontinence, acute stroke, incidence, acute, epidemiology

## Abstract

**Background:** Previously published retrospective series show a high prevalence of fecal incontinence (FI) in stroke patients. We aimed to analyze in a prospective series the current incidence of FI in acute stroke in functionally independent patients and its evolution over time and the patient characteristics associated with the appearance of FI in acute stroke.

**Methods:** We included consecutive patients with acute stroke admitted in our stroke unit who fulfilled the following inclusion criteria: a first episode of stroke, aged >18 years, with no previous functional dependency [modified Rankin Scale (mRS) ≤ 2] and without previous known FI. FI was assessed by a multidisciplinary trained team using dedicated questionnaires at 72 ± 24 h (acute phase) and at 90 ± 15 days (chronic phase). Demographic, medical history, clinical and stroke features, mortality, and mRS at 7 days were collected.

**Results:** Three hundred fifty-nine (48.3%) of 749 patients (mean age 65.9 ± 10, 64% male, 84.1% ischemic) fulfilled the inclusion criteria and were prospectively included during a 20-month period. FI was identified in 23 patients (6.4%) at 72 ± 24 h and in 7 (1.9%) at 90 days ± 15 days after stroke onset. FI was more frequent in hemorrhagic strokes (18 vs. 5%, p 0.007) and in more severe strokes [median National Institute of Health Stroke Scale (NIHSS) 18 (14–22) vs. 5 (3–13), *p* < 0.0001]. No differences were found regarding age, sex, vascular risk factors, or other comorbidities, or affected hemisphere. Patients with NIHSS ≥12 (AUC 0.81, 95% CI 0.71 to 0.89) had a 17-fold increase for the risk of FI (OR 16.9, IC 95% 4.7–60.1) adjusted for covariates.

**Conclusions:** At present, the incidence of FI in acute stroke patients without previous functional dependency is lower than expected, with an association of a more severe and hemorrhagic stroke. Due to its impact on the quality of life, it is necessary to deepen the knowledge of the underlying mechanisms to address therapeutic strategies.

## Introduction

Fecal incontinence (FI) is defined as an involuntary loss of solid or liquid feces. It is a highly prevalent condition in the general population, although its incidence and prevalence are usually underestimated due to reluctance of patients to report its symptoms (1). Large community-based studies have shown a wide variability of rate of FI in the general population ranging from 1 to 24% (2, 3). The maintenance of fecal continence is the result of the integration of somatic pelvic motor coordination and visceral and sensory functions. Therefore, FI may present as a result of an anal sphincter dysfunction, an abnormal rectal compliance, a decreased rectal sensation, or a combination of any of those abnormalities (4, 5).

FI has been reported to be a common complication after stroke. Previous studies have estimated an incidence from 10 to 40% (6–9) according to the time of assessment after stroke (6). In one of the largest epidemiological studies, the prevalence of post-stroke FI ranged from 30% within 10 days to 15% at 3 years after stroke (6), whereas a prevalence of 5 to 6% was detected in two more recent studies beyond the acute phase (10, 11). Additionally, severe FI was reported in 5% of stroke survivors in a large population-wide survey, a four-fold increase compared with non-stroke patients (9).

FI in stroke patients has been associated with age, diabetes mellitus, stroke severity, and other comorbidities (6). Importantly, stroke patients with FI had higher risk of short- and long-term mortality (6) and an increased need for institutionalization and nursing support in the community (10, 11), leading to a healthcare estimated costs of 55% higher compared with stroke patients without FI (12). However, the available published data about FI after stroke are mostly based on studies conducted before the widespread use of intravenous thrombolysis and especially, mechanical thrombectomy in acute stroke treatment, and additionally, FI information has been retrospectively collected through the use of general questionnaires or non-specific clinical scales for FI diagnosis.

Remarkably, the general approach to stroke care has been deeply changing over the last decades. The implementation of dedicated stroke units managed by trained personnel, as well as the focus on rehabilitation strategies, with its facilities and the unequivocal benefit of reperfusion treatments have significantly reduced stroke patient dependency (13). In this new scenario, the aims of this study were to identify (1) the current incidence of FI in acute stroke in previous functionally independent patients, (2) the incidence of persistent FI in stroke patients beyond the acute phase, and, (3) the patient characteristics associated with the appearance of FI in acute stroke.

## Methods

We conducted a prospective observational study of consecutive stroke patients admitted to our stroke unit from May 2018 to December 2019.

### Data Sample

Patients fulfilling the following criteria were included in the study: age ≥18 years, first-ever anterior circulation stroke, functionally independent patients defined as a modified Rankin Scale (mRS) score ≤ 2, and absence of previous FI due to other etiologies. Every included patient was prospectively screened for the presence of new-onset fecal incontinence at 72 ± 24 h (acute phase) after stroke onset and at 90 ± 15 days (chronic phase).

**Figure 1:**
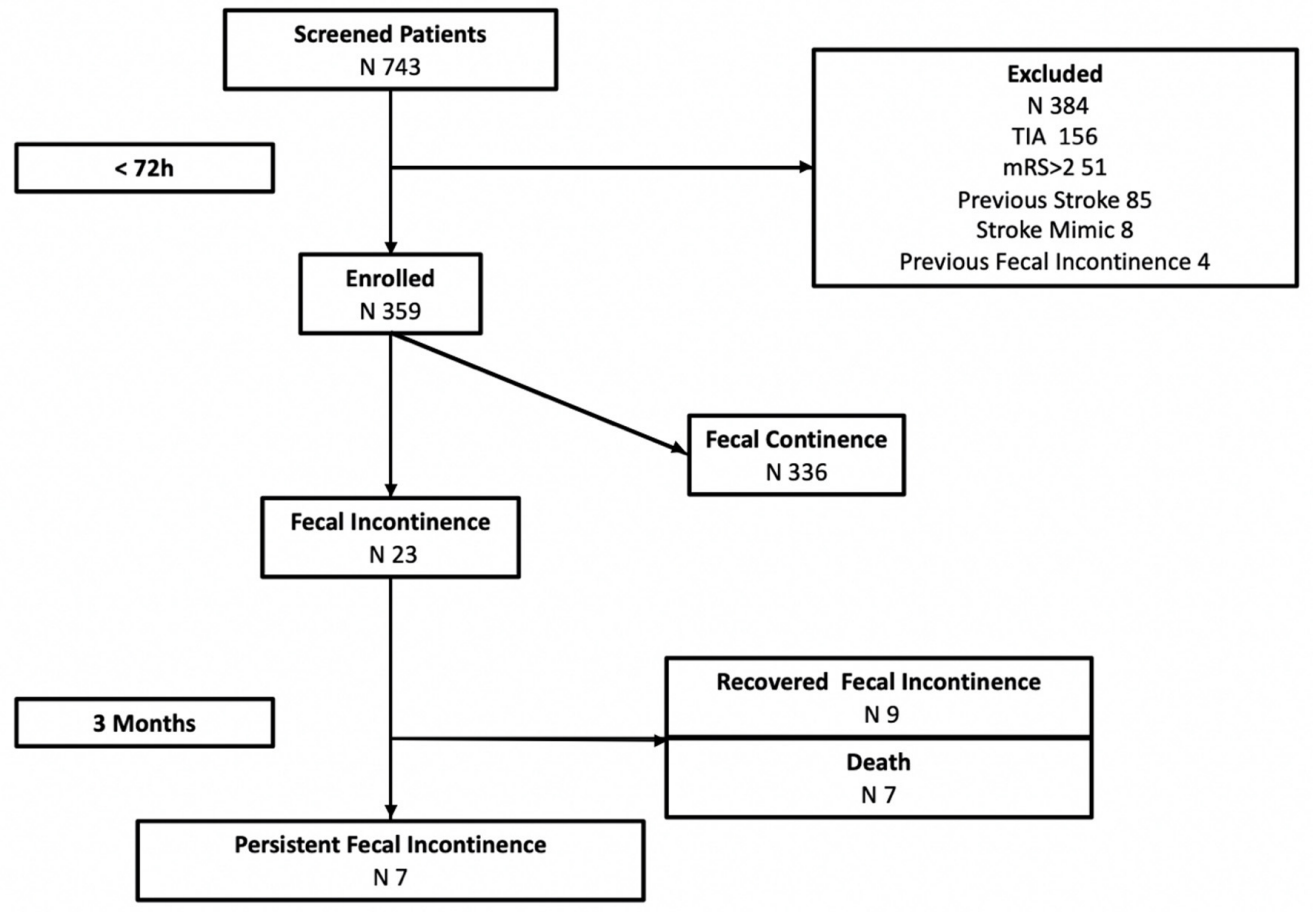
FINISH study workflow. TIA, transient ischemic attack; mRS, modified Rankin Scale.

Out of 743 patients admitted to the stroke unit for a 20-month period, 359 (48.3%) patients (mean age 65.2 ± 10 years, 63.2% male, and 86.2% ischemic stroke) fulfilled the inclusion criteria and were included in the study. Figure 1 shows the flow chart of the study and the reasons to exclude patients.

### Fecal Incontinence Assessment

Data about fecal depositions are routinely collected by our trained stroke unit nurses during hospitalization, and when suspected, FI diagnosis was confirmed by a trained physician both in the acute and chronic phase. FI was defined as any involuntary leakage of feces according to the Rome IV criteria (14), and patients were classified for the purpose of the study in FI and No FI subgroups accordingly. At 3 months follow-up, a dedicated questionnaire [the Wexner score (15)] was administered by a trained surgeon. This questionnaire investigates the presence of uncontrolled loss of gas, liquid, and solid stools, if their presence requires the use of physical restraints such as dressings or diapers and the impact on the quality and sexual life. The score range from the minimum score = 0 which means “perfect continence” to the maximum score = 20 points which means “totally incontinent.”

### Studied Variables

Demographic, previous functional status, vascular risk factors, comorbidities, gastrointestinal tract diseases, history of abdominal surgeries, and stroke characteristics were recorded in a dedicated database. Abdominal surgeries and gastrointestinal diseases were defined as any intervention or relevant pathology involving the gastrointestinal tract previous to stroke.

Stroke severity was defined using the National Institute of Health Stroke Scale (NIHSS) (16). Ischemic stroke etiology was determined according to the Trial of ORG 10172 in acute stroke treatment (TOAST) (17). Functional status was defined by the modified Rankin Scale Score (mRS) at admission and discharge (18). Favorable functional outcome was defined as a mRS ≤ 2 at 3 months.

The study protocol was approved by the local Ethics Committee, and written informed consent was obtained from patients or relatives.

### Statistical Analysis

Descriptive analyses were obtained for all demographic and clinical variables. Baseline characteristics between groups (patients with FI and non-FI) were compared using X^2^ test, Fisher's test, *t*-test, or Mann–Whitney's U-test, as appropriate. Logistic regression analysis was used to determine predictor factors independently associated with the presence of FI in the univariate analysis (*p* < 0.05) and variables previously related to in other published articles. Receiver-operator characteristics (ROC) curves were constructed by plotting basal NIHSS that better predict the presence of FI, according to the best specificity and sensitivity. Statistical significance was defined as two-sided *p*-value <0.05. All the statistical analyses were made using SPSS statistical package vs. 22 (IBM Deutschland GmbH, Ehningen, Germany).

## Results

FI was identified in 23 (6.4%) out of 359 included patients in the acute phase. Of those, at 3 months, seven (30.4%) patients died (three during hospitalization, four after discharge), nine (39.1%) patients recovered FI, and seven (30.4%) patients had persistent FI. Thus, 2% of the first-ever anterior circulation strokes and 44% of stroke patients with FI in the acute phase had persistent FI after stroke.

### Patients Characteristics Associated With Post-stroke Fecal Incontinence

Demographic and clinical data according to the presence of FI are shown in Table 1.

**Table 1:** Baseline clinical characteristics and functional outcome of fecal incontinence and continence stroke patients.

	**Total sample N = 359**	**Fecal incontinence** **N = 23**	**Fecal continence** **N = 336**	***P***
**Demographics and medical history**				
Gender, male	229 (64)	12 (52.1)	217 (64.5)	*0.129*
Age (years)	65.9 ± 10	65.7 ± 10	67.8 ± 11	*0.115*
Smoking habit	90 (25.1)	5 (21.7)	85 (25.3)	*0.639*
Alcohol abuse	25 (6.9)	0 (0)	25 (7.4)	*0.239*
Hypertension	237 (66)	16 (69.5)	221 (65.8)	*0.899*
Diabetes	104 (29)	6 (26.1)	98 (29.2)	*0.919*
Hypercholesterolemia	194 (54)	12 (52.1)	182 (54.2)	*0.766*
Atrial Fibrillation	63 (17.5)	6 (26.1)	57 (16.7)	*0.413*
Heart disease	41 (11.4)	2 (8.6)	39 (11.6)	*0.753*
COPD	36 (10)	3 (13)	33 (9.8)	*0.729*
Chronic kidney failure	9 (2.5)	0 (0)	9 (2.7)	*1.000*
Obesity	42 (11.7)	1 (4.3)	41 (12.2)	*0.271*
Urinary incontinence	0 (0)	0 (0)	0 (0)	*1.000*
Abdominal surgery	28 (7.8)	3 (13)	25 (7.4)	*0.431*
Gastrointestinal disease	18 (5)	1 (4.3)	17 (4.2)	*0.881*
**Clinical features**				
Ischemic stroke Hemorrhagic stroke	302 (84.1) 57 (15.9)	16 (69.5) 7 (20.5)	286 (85.1) 50 (14.9)	*0.008*
Affected side				0.717
Left	200 (55.7)	16 (69.5)	184 (55.3)	
Right	149 (41.5)	7 (30.4)	140 (42)	
Bilateral	9 (2.5)	0 (0)	9 (2.7)	
Arterial occlusion	N = 302	N = 16	N = 286	0.500
No	86 (28.4)	3 (18.7)	83 (29)	
TICA	18 (5.9)	1 (6.2)	17 (5.9)	
ACM M1	158 (44)	6 (37.5)	152 (53.1)	
ACM M2	25 (8.2)	2 (12.5)	23 (8)	
TANDEM	8 (2.6)	2 (12.5)	6 (2)	
ACA	7 (2.3)	2 (12.5)	5 (1.7)	
Reperfusion treatment	N = 302	N = 16	N = 286	0.252
No	162 (53.6)	9 (56.2)	153 (53.1)	
IV tPA	52 (17.2)	0 (0)	52 (18.2)	
EVT	49 (16.2)	4 (25)	45 (15.8)	
IV tPA + EVT	41 (13.6)	3 (18.8)	38 (13.3)	
Intracranial hemorrhage location	N = 57	N = 7	N = 50	0.102
Lobar	22 (38.6)	3 (42.8)	19 (38)	
Basal ganglia	33 (57.9)	4 (57.2)	29 (58)	
Both	2 (3.5)	0 (0)	2 (4)	
TOAST classification	N = 302	N = 16	N = 286	0.152
Atherothrombotic	103 (34.1)	5 (31.5)	98 (34.2)	
Cardioembolic	100 (33.1)	8 (50)	92 (32.1)	
Lacunar	45 (14.9)	0 (0)	45 (15.7)	
Undetermined	39 (12.9)	3 (18.5)	36 (12.5)	
Other causes	15 (5)	0 (0)	15 (5.2)	
NIHSS at baseline	6 [3–6]	18 [14–22]	5 [3–13]	<0.001
NIHSS at 7 days	2 [0–5]	13 [5–21]	2 [0–5]	<0.001
mRS ≤ 2 at 7 days or discharge	182 (50.6)	2 (9)	180 (53.6)	<0.001
Mortality at 7 days or at discharge	11 (3.1)	3 (13)	8 (2.7)	0.029

*Numbers are expressed as mean ± SD, median [quartiles] and number (percentages)*.

*COPD, chronic obstructive pulmonary disease; heart disease, coronary heart disease or heart failure; TICA, terminal internal carotid artery; MCA, middle cerebral artery; ACA, anterior cerebral artery; Tpa, tissue plasminogen activator; EVT, endovascular treatment; TOAST, Trial of ORG 10172 in acute stroke treatment; NIHSS, National Institute of Health Stroke Scale; mRS, modified Rankin Score*.

Patients with FI had higher stroke severity [NIHSS 18 (14–22) vs. 5 (3–13)] at admission compared with patients with fecal continence. FI was more frequent in hemorrhagic stroke (12 vs. 5%) compared with ischemic stroke. No significant differences were found in age, sex, vascular risk factors, history of abdominal surgery, or other comorbidities, affected hemisphere, vessel occlusion, intracranial hemorrhage location, or ischemic stroke etiology between patients with and without FI. At discharge, patients with FI had higher stroke severity, poor functional outcome, and higher mortality rate compared with those without FI (Table 1).

### Independent Predictors of Post-stroke Fecal Incontinence Appearance

**Figure 2:**
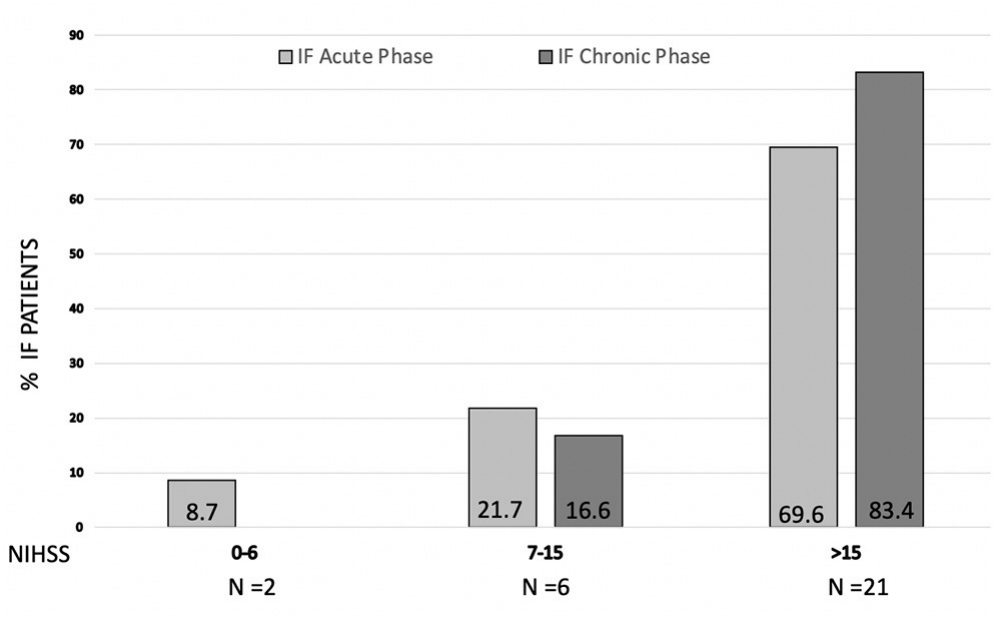
Prevalence of fecal incontinence (FI) in acute phase (gray bar) and chronic phase (black bar) according to stroke severity (NIHSS) at admission. Inside each bar % of FI patients is shown.

A logistic regression model adjusted for age, sex, and affected hemisphere showed that hemorrhagic stroke and NIHSS were independently associated with the presence of FI in the acute phase of stroke (Table 2). A further model adjusted for vascular risk factors or other comorbidities as well did not modify the effect (data not shown). A cut-off point of NIHSS ≥12 in the ROC curve (AUC 0.81, 95% CI 0.71 to 0.89; p < 0.001) predicted the presence of FI with a sensitivity of 84% and specificity of 75%. Stroke patients with NIHSS≥ 12 had a 17-fold increase in the risk of FI compared with those with NIHSS <12 (OR 16.9 IC 95% 4.7–60.1) adjusted for covariates. Moreover, when we classified stroke patients according to stroke severity in mild stroke (NIHSS ≤ 6), moderate stroke (NIHSS 7–15), and severe stroke (NIHSS>15) (19), we found that the higher the stroke severity, the higher the rate of FI in the acute phase and in the chronic phase (Figure 2).

**Table 2:** Multivariate analysis of factors associated with fecal incontinence (FI) during the acute stroke phase.

	**OR**	**95% IC**	**p**
Age	1.010	0.967–1.056	0.65
Female gender	2.308	0.876–6.084	0.091
Left side stroke	1.629	0.613–4.327	0.328
Hemorrhagic stroke	4.743	1.701–13.224	0.003
NIHSS admission >12	16.915	4.759–60.120	0.001

Table 3 shows the patient and stroke characteristics associated with persistent FI at 3 months. No differences were found in clinical, demographic features, stroke subtype, baseline NIHSS, and severity of FI measured by the Wexner's score between patients with persistent FI or recovered FI at 3 months. Thus, no independent predictors of persistent FI beyond acute stroke phase were identified in a logistic regression analysis (data not shown).

**Table 3:** Baseline clinical characteristics of patients with persistent fecal incontinence and recovered fecal incontinence at 90 days.

	**Total sample *N* = 16**	**Persistent fecal Incontinence *N* = 7**	**Recovered fecal incontinence** ***N* = 9**
**Demographics and medical history**			
Gender, male	6 (37.5)	2 (28.5)	4 (44.4)
Age (years)	66.4 ± 12.4	69.7 ± 10.7	62.7 ± 18.2
Smoker	3 (18.7)	1 (14.3)	2 (22.2)
Alcohol abuse	0 (0)	0 (0)	0 (0)
Hypertension	6 (37.5)	1 (14.3)	5 (55.5)
Diabetes	4 (25)	3 (42.9)	1 (11.1)
Hypercholesterolemia	6 (37.5)	3 (42.9)	3 (33.3)
Atrial fibrillation	1 (6.2)	0 (0)	1 (11.1)
Heart disease	2 (12.5)	2 (28.6)	0 (0)
COPD	1 (6.2)	1 (14.3)	0 (0)
Chronic kidney failure	0 (0)	0 (0)	0 (0)
Obesity	1 (6.2)	1 (13.3)	0 (0)
Urinary incontinence	0 (0)	0 (0)	0 (0)
Abdominal surgery	3 (18.7)	2 (28.6)	1 (11.1)
Gastrointestinal disease	1 (6.2)	1 (14.3)	0 (0)
Clinical features			
Ischemic stroke Hemorrhagic stroke	10 (62.5) 6 (37.5)	4 (57.1) 3 (42.9)	6 (66.7) 3 (33.3)
Side			
Left Right Bilateral	9 (56.2) 7 (43.7) 0 (0)	4 (57.1) 3 (42.94) 0 (0)	5 (55.6) 4 (44.4) 0 (0)
Arterial occlusion No TICA ACM M1 ACM M2 TANDEM ACA	*N* = 10 1 (10) 0 (0) 5 (50) 1 (10) 1 (10) 2 (20)	*N* = 4 0(0) 0(0) 3(75) 1 (25) 0 (0) 0 (0)	*N* = 6 1 (16.7) 0 (0) 2 (33.3) 0 (0) 1 (16.7) 2 (33.3)
Hemorrhage localization Lobar Basal ganglia Both	*N* = 6 3 (50) 3 (50) 0 (0)	*N* = 3 3 (100) 0 (0) 0 (0)	*N* = 3 0 (0) 3 (100) 0 (0)
TOAST classification Atherothrombotic Cardioembolic Lacunar Undetermined Incomplete	*N* = 10 3 (30) 5 (50) 0 (0) 2 (20) 0 (0)	*N* = 4 2(50) 1(25) 0(0) 1(25) 0 (0)	*N* = 6 1 (16.7) 4 (66.7) 0 (0) 1 (16.7) 0 (0)
NIHSS at baseline	18 [17–20]	18 [17–21]	19 [9–21]
Wexner Score	9 [8–12]	10 [8–12]	9 [8–13]

*Numbers are expressed as mean ± SD, median [quartiles] and number (proportion)*.

*COPD, chronic obstructive pulmonary disease; heart disease, coronary heart disease or heart failure; TICA, terminal internal carotid artery; MCA, middle cerebral artery; ACA, anterior cerebral artery; tPA, tissue plasminogen activator; EVT, endovascular treatment; TOAST, Trial of ORG 10172 in acute stroke treatment; NIHSS, National Institute of Health Stroke Scale*.

*The p-values are not shown due to no statistical significance*.

## Discussion

Stroke patients may suffer different complications both during the acute (such as neurological worsening or stroke recurrence, seizure, infections) and the chronic phase (cognitive impairment, depression, and emotional lability, among others) (20). Fecal incontinence in stroke patients has been poorly investigated during the last decade, probably due to a lack of interest of neurologists and its main prevalence in long-term hospitalized stroke patients. However, FI has a strong impact on a person quality of life and on its caregiver (21).

The present study shows an incidence of 6% of FI in first-ever functionally independent stroke patients during the acute phase that decreases to 2% at 3 months. This rate is significantly lower than expected and reported before. Brocklehurst et al. (22) observed FI in 23% of patients immediately after stroke, which diminished down to 16% after 2 weeks, and eventually to 8% at 1 year. The large registry of the Copenhagen Stroke study (23) reported FI in 40% of stroke patients at hospital admission, in 18% at hospital discharge, and in 9% at 6 months. Of note, the Harari et al. (6) study showed that the prevalence of FI was of 30% at 7 to 10 days, 11% at 3 months, 11% at 1 year and 15% at 3 years. Recently, Jacob et al. (24) showed that up to 6% of stroke survivors presented FI at some point during the 10-year study period in a large retrospective cohort. It is important to highlight that these studies identified FI through the use of Barthel Index scale or a customized questionnaire (9, 11), and no other specific or dedicated scales, or clinical or electrophysiological assessments were done to properly diagnose FI. We selected the Wexner score (15) because it is a widely used scale in fecal incontinent patients, usually employed by proctologists and gastroenterologists to evaluate fecal incontinence and its severity. It has been widely validated in FI assessment and it strongly correlates with FI severity. Wexner score has been related to subjective perception of FI and it could be self-assessed by patients. This fact and the retrospective evaluation of some of these previous studies could partly explain the difference in the incidence rates with respect to our series, in which the patients were prospectively assessed with scales designed to the detection of FI. In addition and importantly, the improvement in stroke care through its management in stroke units and the advancement in reperfusion treatments, rehabilitation strategies, and patient and caregiver education, have played an important role on the functional outcome of stroke patients in the last decade (25), and it could have strongly modified the incidence and the natural history of fecal incontinence in acute stroke.

Moreover, It is known that the lack of mobility, the functional dependence, and indeed, the use of multiple medications are risk factors to develop fecal incontinence (6, 10, 25, 26). One of the pillars of stroke rehabilitation is the early mobilization and the implementation of instrument use to improve the self-care and autonomy of the patient, so, this approach could as well have reduced the incidence of FI during the last years. Nevertheless, 30% of patients with FI detected during the acute phase in our cohort died before 90-days follow-up evaluation, so we cannot rule out a potential higher incidence of persistent FI after stroke (up to 3.8% higher).

According to our results, the strongest independent predictor to have FI was stroke severity at admission, since patients with a severe stroke (NIHSS >12) have a 17-fold increase in the probability of developing FI in the acute phase compared with patients with NIHSS ≤ 12 (Figure 2). Harari et al. (6) already described the association between stroke severity and FI although using only clinical features (neglect, dysphagia, etc.) instead of a validated and widely used stroke scale for severity assessment such as NIHSS (16). In our cohort, patients suffering from a hemorrhagic stroke were five times more likely to present FI during the acute phase compared with patients with ischemic stroke, which was not previously found associated with FI. The limited acute treatment strategies able to modify the natural course and functional outcome of intracranial hemorrhage and, as already described, the more severe disability that usually implies hemorrhagic stroke compared with ischemic stroke (27) [median NIHSS 20 (15–23) vs. 18 (14–22) in hemorrhagic/ischemic stroke in our series], might explain the higher incidence of FI in these patients. Interestingly, no association has been found with vascular risk factors, deliveries, and previous abdominal surgeries that have been already related to FI (6). Although it could be due to the sample size, we infer that this could strengthen the role of the acute stroke as the primary cause of FI appearance.

To the best of our knowledge, this is the first study specifically designed to prospectively investigate the appearance of FI using a multidisciplinary approach. We implemented a comprehensive protocol that allowed us to study our patients by means of the stroke neurologist and the proctologist. We used the Wexner scale instead of the Barthel index to quantify fecal incontinence in the chronic phase that provides a deeper description of the FI compared with the dichotomic item within the functional clinical assessment of the Barthel Index. Our results show a moderate severity of FI in our patients (Wexner score 9–10).

Remarkably, half of the incontinent patients in the acute phase of stroke were still affected 3 months after stroke, although no differences were found in clinical or demographic features between those patients with persistent or recovered FI. Of note, the severity of the FI diagnosed in the acute phase did not correlate with its persistence at 3 months follow-up.

Interestingly, the cerebral mechanisms underlying the appearance of FI in stroke patients are not completely understood (28–31). The maintenance of fecal continence is the result of a coordination of pelvic motor, visceral and sensory functions; therefore, the loss of fecal continence may be the result of an anal sphincter dysfunction, an abnormal rectal compliance, a decrease rectal sensation, or a combination of any of these abnormalities (5, 32, 33). We found that FI was linked to a more severe stroke, with a probable large hemispheric lesion, and contrarily, no association was found to stroke lateralization. Until now, there is no knowledge about the precise cortical area involved in the fecal continence (the “continence” area), so, further anatomical and radiological functional studies are needed.

The main strengths of this study are the high-quality data due to the systematic and prospective data collection, the multidisciplinary approach, the use of FI specific clinical scales and stroke severity assessment. Thus, it has followed a large sample of patients in an observational study in a short period of time without differences in the acute stroke care, recanalization techniques, and rehabilitation facilities for a long time between patients. However, several limitations have to be stated. First, our study was carried out in a single comprehensive tertiary stroke center, so these results might not be generalizable to other settings. Second, we focused on the incidence of FI in the acute phase until 3 months after stroke. Consequently, we did not assess the potential resolution or the new appearance of FI beyond this period. Third, we excluded patients with previous stroke and/or other gastrointestinal tract disease to select patients in whom the acute stroke was the single cause of FI, a fact that could contribute to rule out patients in whom FI was multifactorial. Fourth, we did not specifically collect language impairment or cognitive disturbance, which might have altered the continence independently of the stroke; however, we included only functionally independent patients (mRS < 2), able to live alone and to carry on daily life activities by themselves. So, we think that the cognitive impairment, if present, would have been mild or very mild. Last, we also excluded patients with vertebrobasilar stroke territory to minimize clinical heterogeneity in the sample of patients, so a higher incidence of FI could have been identified.

Sacral nerve stimulation is an established treatment for FI due to several etiologies (34). Its neuromodulatory effect on cortical areas would be promising for incontinent patients due to stroke in addition with standard care based on rehabilitation.

Therefore, prospective randomized clinical studies analyzing the real effect of neuromodulation for FI in stroke patients are warranted.

## Conclusion

At present, the incidence of persistent fecal incontinence reaches 2% of the previous functionally independent patients with a first anterior circulation stroke, lower than expected and strongly related to stroke severity. However, due to its impact on the quality of life, it is necessary to deepen the knowledge of the underlying mechanisms to address therapeutic strategies.

## Data Availability Statement

The raw data supporting the conclusions of this article will be made available by the authors, without undue reservation.

## Ethics Statement

The studies involving human participants were reviewed and approved by the Ethic Committee of our center - CEIC (comité ético de Investigación Hospital Germans Trias i Pujol). The patients/participants provided their written informed consent to participate in this study.

## Author Contributions

GL, DP, MM, JC, JS, JB, LR-E, MA, and AM-P designed the study. JC, JB, AP, MH-P, MA, and AM-P selected the patients and included the subject. SC, MH-P, HO, and SD revised the patients included. GL, MM, and DP wrote and revised the manuscript. All authors contributed to the article and approved the submitted version.

## Funding

This project was granted by Fundació/Marató TV3 (ID Number: 201718.10). MM and MH-P are part of the research group RETICS-INVICTUS-PLUS (RD0016/0019/0020) granted from the Instituto de Salud Carlos III (Spanish Ministry of Economy and Competiveness). MH-P receives funding from Instituto de Salud Carlos III with a grant for Health Research (JR17/00006).

## Conflict of Interest

The authors declare that the research was conducted in the absence of any commercial or financial relationships that could be construed as a potential conflict of interest.

## Publisher's Note

All claims expressed in this article are solely those of the authors and do not necessarily represent those of their affiliated organizations, or those of the publisher, the editors and the reviewers. Any product that may be evaluated in this article, or claim that may be made by its manufacturer, is not guaranteed or endorsed by the publisher.
